# T Cell Control of Extracellular Matrix Degradation

**DOI:** 10.1155/2000/43657

**Published:** 2000

**Authors:** Yves St-Pierre, Edouard E. Potworowski

**Affiliations:** 1 Centre de Recherche en Santé Humaine INRS-Institut Armand-Frappier Université du Quebec Laval Canada; 2 INRS-Institut Armand-Frappier 531 Boul. des Prairies Laval Qurbec H7V 1B7 Canada

**Keywords:** Matrix metalloproteinases (MMP), Extracellular matrix (ECM), Tissue Inhibitor of MMP (TIMP), adhesion molecules


*Developmental Immunology*, 2000, Vol. 7(2-4), pp. 171-177
Reprints available directly from the publisher
Photocopying permitted by license only

© 2000 OPA (Overseas Publishers Association)
N.V. Published by license under the
Harwood Academic Publishers imprint,
part of the Gordon and Breach Publishing Group.
Printed in Malaysia

### T Cell Control of Extracellular Matrix Degradation

YVES ST-PIERRE* and EDOUARD F. POTWOROWSKI

*Centre de Recherche en Santé Humaine, INRS-Institut Armand-Frappier, Université du Quebec, Laval, Canada*

*Keywords:* Matrix metalloproteinases (MMP), Extracellular matrix (ECM), Tissue Inhibitor of MMP (TIMP), adhesion molecules

### INTRODUCTION

Matrix metalloproteinases (MMP) are members of an enzyme family the extent of whose involvement in physiological and pathological situations is just beginning to be appreciated. A case in point is the vast array of normal and exacerbated immunological responses involving T cell recruitment to a particular site in the organism through the extracellular matrix (ECM). In order to penetrate and migrate through the ECM, T cells need to lyse their way through a variety of connective tissue proteins. We review herein the available evidence for a role of MMPs secreted by T cells to allow them to migrate. We will then examine the data indicating that MMPs are not solely secreted by migrating T cells but that adjacent stromal cells also do their part, Finally, we will review a number of instances where MMP dysregulation is associated with some important T-cell dependent pathologies.

### EXPRESSION OF MMP BY T CELLS WILL DICTATE THEIR CAPACITY TO MIGRATE THROUGH EXTRACELLULAR MATRIX

MMPs are zinc-dependent enzymes and include collagenases, gelatinases A and B, the stromelysins, matrylisins, metalloelastase, and the membrane-type metalloproteinases (Massova et al., 1998). Most of the members of the MMP family are secreted by cells into the extracellular space; nonetheless, some membrane-type MMPs (MT-MMP) attached to the cell surface have also been described (Sato et al., 1994, Strongin et al., 1995, Matsumoto et al., 1997, Shofuda et al., 1997, Pei, 1999), and shown, like their secreted counterparts, to participate in the enzymatic cascade that leads to the degradation of proteins of the ECM.

Circulating T cells in a resting state express a very limited repertoire of MMPs. Constitutive expression of only MT1-MMP, MMP-2, and MMP-9 have been reported to be expressed in resting T cells (Weeks et al., 1993, Leppert et al., 1995, Graesser et al., 1998). It is the recruitment of T cells to the inflamed endothelium via secretion of chemokines that initiates a cascade of activation events leading to both the expansion of the MMP repertoire by T cells, as well and the increase of the expression level of specific MMPs. Chemokines that are released locally during an inflammatory response, such as RANTES, VIP, and MIP, are likely the first modulators that initiate the expansion of the MMP repertoire in T cells, judging from their ability to induce *in vitro* the expression of MMP-9 in T cells (Xia et al., 1996). Although

* Correspondence to: INRS-Institut Armand-Frappier, 531 Boul. des Prairies, Laval, Québec, Canada H7V 1B7. Phone: 450–686–5354, Fax: 450–686–5501, E-mail: yves.st-pierre@inrs-iaf.uquebec.ca binding of L-selectin on the surface of neutrophils can also induce neutrophils to secrete MMP-9 (Wize et al., 1998), there is as yet no indication that the repertoire of MMPs can be modulated during the rolling of T lymphocytes on the inflamed endothelium. In contrast, firm contact between circulating T cells and the endothelial cells via cell adhesion molecules is important for the induction of MMP genes in T cells; we have shown that upregulation of MMP-9 required direct contact with endothelial cells, since antibodies to LFA-1 and ICAM-1 blocked such an upregulation (Aoudjit et al., 1998). In this particular case, the binding to ICAM-1 was essential but not sufficient to induce MMP-9 in T lymphoma cells. In other models, however, signaling via integrins alone was sufficient to induce MMP genes, as VCAM-1-induced the expression of MMP-1 in T cells (Leppert et al., 1995). Romanic et al. (1994) have also demonstrated that MMP-2 was induced in T cells by adhesion to endothelial cells through the binding of T cell VLA-4 to VCAM-1. It is hypothesized that the induction of MMP-2 by autoreactive T-cell clones via VLA-4 is necessary for the clones to induce experimental autoimmune encephalomyelitis (EAE), a mouse model for human multiple sclerosis (Graesser et al., 1998). Thus, the sequence of the initial events is as follows: 1) inflammatory cytokines produced at the extracellular inflammatory site reachs the vascular endothelium, 2) the endothelium becomes "inflamed", induces and expresses CAMs, such as ICAM-1 and VCAM-1, 3) circulating T cells via integrins adhere to the inflamed endothelium, 4) activation of MMP genes occurs in T cells, and 5) secretion of MMPs confers to T cells an invasive behavior, allowing them to migrate across the vascular wall and deep in the tissue. This is consistent with the idea that memory T cells, which express high levels of VLA-4 and/or LFA-1 on their surface, have an increased capacity to cross the basal lamina and penetrate the tissue stroma. The intracellular signaling pathways that lead to MMP expression in T cells have only been partially characterized, but possibly involve PKC-mediated signaling pathways, as PKC activators have been shown to induce in T cells the expression of MMP-9, MMP-10, and MMP-13 (Weeks et al., 1993, Zhou et al., 1993, Conca and Wilmroth, 1994, Aoudjit et al., 1997, Wilmroth et al., 1998). The development of a repertoire of MMPs by T cells is therefore a dynamic process that is initiated during the first steps of the cascade of adhesion molecule interactions.

### T CELL-DEPENDENT INDUCTION OF MMP IN STROMAL CELLS

The source of MMPs at inflammatory sites is not, however, restricted to the secretion by infiltrating cells: indeed stromal cells themselves can secrete MMPs on contact with infiltrating cells. This idea has emerged from an increasing number of studies in tumor models. Although MMP secretion was initially associated with metastatic cells, *in situ* hybridization has shown that normal cells surrounding the tumors can also secrete MMPs. Histological examination of tumor-containing tissues in patients with squamous cell carcinomas and malignant epithelium have shown that MMP mRNA was expressed not only by tumor cells, but also by normal peritumoral cells as well (Pyke et al., 1992, Basset et al., 1997). In some cases, the expression of MMP-9 was exclusively found in the stroma surrounding the tumors (Karelina et al., 1993). In was indeed shown that contact with several types of tumor cells *in vitro* induced expression of MMP-9 in fibroblasts (Himelstein et al., 1994, Miyagi et al., 1995, Aoudjit et al., 1997), and a new member of the MMP family, stromelysin-3, was identified specifically in stromal cells surrounding invasive breast carcinomas (Basset et al., 1990). Fibroblasts surrounding human breast carcinoma cells have also been implicated in invasion and/or metastasis through the production of MT-MMP, which is thought to facilitate, via activation of MMP-2, the transition of human breast carcinoma cells from a fibroblast- like phenotype to an epithelial-mesenchymal tumorigenic state (Gilles et al., 1997). The importance of MMP production by stromal cells was further emphasized by the same group who showed, using a co-implantation assay, that fibroblasts of stromelysin-3-deficient mice had lost the capacity to promote implantation of MCF-7 human malignant epithelial cells in nude mice (Masson et al., 1998). Although the mechanism by which MMPs enhance tumor growth is yet unclear, there are several indications that such overexpression of MMP could act by stimulating angiogenesis. The recent report by Werb's group in which MMP-9-deficient mice exhibited an abnormal pattern of skeletal growth plate vascularization and ossification due to defective angiogenesis, strongly argues in favor of this possibility (Vu et al., 1998).

There is increasing evidence that such an induction of MMP secretion by stromal cells can also be operative when circulating T cells migrate to an inflammatory site, most notably when they first encounter the vascular endothelium. In this context, our group has shown that T lymphoma cells could induce MMP-9 expression in endothelial cells by TNFα (membrane-bound or soluble) (Aoudjit et al., 1998). This cytokine can also induce other members of the MMP family, such as MT1-MMP (Rajavashisth et al., 1999), while other cytokines, such as TGFβ1, can upregulate MMP-2 and MMP-9 in endothelial cells (Puyraimond et al., 1999). Adhesion molecules on T cells, most notably the CD40 ligand, can also promote the expression of MMPs in endothelial cells, thereby contributing to the vascular remodeling and the neoformation of vessels during atherogenesis and other chronic immune reactions (Mach et al., 1999). The capacity of T cells to induce the secretion of MMPs in stromal cells via CD40/CD40L continues even after the cells have crossed the vascular cell wall and penetrated the tissue stroma; this is shown by the fact that they can induce the expression of MMP-1, MMP-2, MMP-3, and MMP-9 in vascular smooth muscle cells (Schonbeck et al., 1997), thereby supporting the development or maintenance of chronic inflammatory lesions. Isolated membranes from T cell lines expressing high levels of the CD40 ligand also stimulate the expression of mRNA and the production of MMP-9 by human monocytic cells (Malik N et al., 1996). Upregulation of MMP gene expression in stromal cells is, however, cell-type specific: contact of T cells via the CD40/CD40L pathway with gingival fibroblasts inhibits their constitutive or induced production of MMP-1 and MMP-3 (Wassenaar A et al., 1999), a mechanism thought to delay further periodontal tissue damage during chronic periodontitis. These results establish the CD40/CD40L as an important pathway regulating the induction of MMPs in stromal cells upon contact with T cells.

### THE DELICATE BALANCE BETWEEN MMP AND THEIR NATURAL INHIBITORS

The balance between matrix formation and destruction is maintained by the presence of naturally occurring tissue inhibitors of metalloproteinases (TIMPs). Four members of the TIMP family, designated as TIMP-1 to –4, have been identified to date (Denhardt et al., 1993, Greene et al., 1993, Apte et al., 1994, Leco et al., 1994, Silbiger et al., 1994, Leco et al., 1997). TIMPs have a molecular weight of approximately 25–30 kDa and bind to both latent and active MMPs through strong interactions with the hemopexin- like domain of the enzyme, forming a stable and catalytically inert, 1:1 stoichiometric enzyme-inhibitor complex that inhibits activation of the proenzyme in physiologic conditions (Gomis-Ruth *et al.*, 1997).

T lymphocytes are among the cells that have been shown to secrete a restricted repertoire of TIMP genes; TIMP-2 is often expressed constitutively, whereas TIMP-1 is inducible by activation with various stimuli, such as PKC activators. To date, there is no evidence that, in T cells, TIMP-3 or TIMP-4 are expressed constitutively, or upon activation. TIMP-4-specific transcripts are expressed abundantly in the heart, moderately in tissues such as kidneys, colon, testis, ovary, and skeletal muscles, and not at all in the thymus and the spleen (Greene et al., 1996, Leco et al., 1997). Abundant transcripts encoding TIMP-3 were also detected in kidneys, lungs, and brain, but not in lymphoid tissues. In contrast to TIMP-2 and TIMP-4, however, TIMP-3, like TIMP-1, is encoded by an inducible gene, most notably upon exposure to T cell-dependent factors, as its expression is stimulated by AP-1 and NF-kB activators, by 12-O-tetradecanoylphorbol-13-acetate, and by TNFα (Sun et al, 1995). The consequences of T cell-induced modulation of TIMP secretion often vary according to the T cell factor implicated and the target cells; no systematic study, however, has been done and only anecdotal data are available. For instance, whereas TIMP-1 is upregulated in monocytes, synoviocytes, uterine cervical fibroblasts, endothelial cells, and lung fibroblasts upon exposure to IL-1 or IL-6, the same TIMP-1 is downregulated when bronchial epithelial cells are treated with TNFα (Yao et al., 1997). Decreased TIMP-3 secretion in synovial lining cells has also been reported upon exposure to Oncostatin M (Gatsios et al., 1996). The decrease in TIMP expression or the increase in MMP production will thus necessarily tip the balance in favor of enhanced metalloproteinase activity, leading to ECM turnover, and tissue remodeling.

Like other proteins, TIMP:MMP complexes formed in the extracellular space are sensitive to proteolysis by extracellular proteases; moreover, in some cases, partial proteolysis of the complex can free TIMP from the complex, release active MMP, and shift the balance toward ECM proteolysis. One of the most potent enzymes for carrying out this task is the neutrophil elastase (NE), a protease which is abundantly secreted following massive neutrophil infiltration. NE can degrade into small fragments the inhibitory TIMP-1 molecule (Okada et al., 1988), even if it is bound to MMP-9, thereby freeing MMP-9 and shifting the balance toward ECM proteolysis (Itoh et al., 1995). NE is not normally expressed in T cells, although elastolytic activity has been reported to be associated with the T cell receptor complex in some T cell lines (Bristow and Flood, 1993) and malignant T cell lymphomas (Kossakowska et al., 1998). To further complicate matter, NE can indirectly induce MMP secretion by T cells and stromal cells since fragmentation of ECM proteins, such as laminin, exposes TNFα binding sites on these fragments. This not only serves to restrict TNFα to sites adjacent to the inflammation, but possibly induce MMP secretion via binding of CD4-positive T cells to these templates (Hershkoviz et al., 1995). Given the fact that NE further participates in the connective tissue destruction at inflammatory sites by its direct action on elastin and other ECM proteins, NE must be considered as a key participant in the tissue remodeling by MMPs at inflammatory sites.

### DYSREGULATION OF MMP SECRETION IN T CELL-MEDIATED INFLAMMATORY PROCESSES

It is no surprise that MMP secretion is, in most cases, transient and highly regulated, since abnormal secretion of MMPs may be highly detrimental, most notably by favoring angiogenesis and tumor metastasis. The capacity of cancer cells to invade the stroma is attributed to their ability to secrete MMPs capable of degrading the extracellular matrix of basement membranes (reviewed by Powell and Matrisian, 1997). To date, the best *in vivo* evidence for the implication of MMPs in neoplasia comes from gene transfer experiments using experimental models of metastasis in which tumor cells were transfected with vectors encoding TIMP or MMP genes, or inhibitory antisense sequences. Whereas transfection of TIMP genes or of MMP antisense into tumor cells inhibited tumor growth (Hua and Muschel, 1996; Wang et al., 1997; Seghal et al., 1998, Valente et al., 1998), expression of MMPs in non-metastatic cells by gene transfection rendered them capable of disseminating and forming tumors at distant sites (Powell et al., 1993; Bernhardt et al., 1994; Tsunezuka et al., 1996). The expression of TIMPs in transgenic mice has also been shown to exert a suppressive effect on metastasizing tumor, most notably in the case of brain fibrosarcoma (Kruger A, et al., 1998), and lymphoma (Kruger et al., 1997).

There is increasing evidence that an imbalance between MMPs and the associated TIMPs can play a decisive role in several degenerative diseases with an autoimmune etiology, such as rheumatoid arthritis, and multiple sclerosis (MS). MS is characterized by massive release of extracellular proteases. The most common forms of proteases released in extracellular environment include NE, cathepsins, and MMPs. Immunohistochemical studies of the MS brain tissue suggested that monocytes, macrophages and reactive astrocytes are potential sources of increased Cathepsin B levels in MS brain (Bever and Garver, 1995). The most common protease associated with MS, however, is MMP-9 (Gijbels et al., 1993). Analysis of cerebrospinal fluids samples from 52 patients with relapsing-remitting and primary progressive MS showed abnormally high levels of MMP-9 (Leppert et al., 1998). This increase was found even in clinically stable patients, suggesting that progressive tissue damage results from an ongoing proteolysis by MMP-9. *A priori*, the role played by proteases in chronic inflammation and autoimmunity consists of cleaving ECM proteins, such as collagen, thereby disrupting the blood brain barrier and facilitating the infiltration of effector cells, most notably T cells, monocytes, PMN, and NK cells. In the case of MS, the ability of proteases to degrade myelin basic protein (MBP) may be crucial (Gijbels et al., 1993). Not only does the degradation of the myelin sheath damage neuronal functions of the CNS, it also generates proteolytic fragments that contribute to local auto-immune reaction against MBP antigens. The positions of the cleavage sites in human MBP following degradation by MMP-9 is such that at least one peptide coincides with a documented major MBP-autoantigen (Proost et al., 1993). Other MMPs, such as matrilysin (MMP-7) can also degrade MBP; indeed an injection of MMP-7 and MMP-9 into the brain parenchyma induces loss of myelin integrity (Anthony et al., 1998). Cathepsins are also capable of degrading MBP, thereby contributing to the release of potential MBP auto-antigens (Cao et al., 1999). The efficacy of IFNβ treatment in MS patients may be in part due to the ability of this drug to decrease MMP-9 activity, leading to a reduction of T-lymphocyte infiltration into the CNS (Leppert et al., 1996, Stuve et al., 1996).

Selective inhibition of MMPs could be a useful approach to counter the progression of autoimmune degenerative disorders where extensive tissue remodeling is present. There is an increasing number of ongoing clinical trials focusing on the potential benefit of MMP inhibitors (Parsons SL, et al. 1997, Beattie GJ, et al. 1998, Bramhall SR, 1998, Wojtowicz-Praga S et al., 1998, Brown PD, 1999, Primrose JN et al., 1999). These trials have so far been restricted mostly to the field of cancer, where MMPs have been shown to favor angiogenesis and confer invasive behavior to metastatic cells (Bernhard et al., 1994). However, with a better understanding of the role of MMPs in the immune response and disorders, it is likely that MMP inhibitors will be used in the treatment of chronic autoimmune disorders where ongoing proteolysis and degenerative tissue remodeling occur.

### *Acknowledgements*

Supported by the Medical Research Council of Canada, and the Fond pour la Formation de chercheur et d'Aide à la Recherche (FCAR).

### *References*

Anthony D.C., Miller K.M., Fearn S., Townsend M.J., Opdenakker G., Wells G.M., Clements J.M., Chandler S., Gearing A.J., and Perry V.H. (1998). Matrix metalloproteinase expression in an experimentally-induced DTH model of multiple sclerosis in the rat CNS. J Neuroimmunol. 87:62–72.

Aoudjit F., Esteve P.O., Desrosiers M., Potworowski E.F., and St-Pierre Y. (1997). Gelatinase B (MMP-9) production and expression by stromal cells in the normal and adult thymus and experimental thymic lymphoma. Int J Cancer. 71:71–8.

Aoudjit F., Potworowski E.F., and St-Pierre Y. (1998). Bi-directional induction of matrix metalloproteinase-9 and tissue inhibitor of matrix metalloproteinase-1 during T lymphoma/endothelial cell contact: implication of ICAM-1. J Immunol. 160:2967–73.

Apte S.S., Mattei M.G., and Olsen B.R. (1994). Cloning of the cDNA encoding human tissue inhibitor of metalloproteinases-3 (TIMP-3) and mapping of the TIMP3 gene to chromosome 22. Genomics 19:86–90.

Basset P., Bellocq J.P., Wolf C., Stoll I., Hutin P., Limacher J.M., Podhajcer O.L., Chenard M.P., Rio M.C., and Chambon P. (1990). A novel metalloproteinase gene specifically expressed in stromal cells of breast carcinomas. Nature 348:699–704.

Basset P., Okada A., Chenard M.P., Kannan R., Stoll I., Anglard P., Bellocq J.P., and Rio M.C. (1997). Matrix metalloproteinases as stromal effectors of human carcinoma progression: therapeutic implications. Matrix Biol. 15:535–41.

Beattie G.J., and Smyth J.F. (1998). Phase I study of intraperitoneal metalloproteinase inhibitor BB94 in patients with malignant ascites. Clin Cancer Res. 4:1899–902.

Bernhard E.J., Gruber S.B., and Muschel R.J. (1994). Direct evidence linking expression of matrix metalloproteinase 9 (92-kDa gelatinase/collagenase) to the metastatic phenotype in transformed rat embryo cells. Proc Natl Acad Sci U S A. 91:4293–7.

Bever C.T. Jr, and Garver D.W. (1995). Increased cathepsin B activity in multiple sclerosis brain. J Neurol Sci. 131: 71–73.

Bramhall S.R. (1998). Stromal degradation by the malignant epithelium in pancreatic cancer and the therapeutic potential of proteolytic inhibition. J Hepatobiliary Pancreat Surg. 5:392–401.

Bristow C.L., and Flood P.M. (1993). T cell antigen receptor immune complexes demonstrating biologic and proteolytic activity. Int Immunol. 5:79–88.

Brown P.D. (1999). Clinical studies with matrix metalloproteinase inhibitors. APMIS. 107:174–80.

Cao L., Goodin R., Wood D., Moscarello M.A., and Whitaker J.N. (1999). Rapid release and unusual stability of immunodominant peptide 45–89 from citrullinated myelin basic protein. Biochemistry 38:6157–63.

Conca W., and Willmroth F. (1994). Human T lymphocytes express a member of the Matrix Metalloproteinase gene family. Arthritis Rheum. 37:951–6.

Denhardt D.T., Feng B., Edwards D.R., Cocuzzi E.T., and Malyankar U.M. (1993). Tissue inhibitor of metalloproteinases (TIMP, aka EPA): structure, control of expression and biological functions. Pharmacol Ther. 59:329–41.

Gatsios P., Haubeck H.D., Van de Leur E., Frisch W., Apte S.S., Greiling H., Heinrich P.C., and Graeve L. (1996). Oncostatin M differentially regulates tissue inhibitors of metalloproteinases TIMP-1 and TIMP-3 gene expression in human synovial lining cells. Eur J Biochem. 241:56–63.

Gijbels K., Proost P., Masure S., Carton H., Billiau A., and Opdenakker G. (1993). Gelatinase B is present in the cerebrospinal fluid during experimental autoimmune encephalomyelitis and cleaves myelin basic protein. J Neurosci Res. 36:432–40.

Gilles C., Polette M., Seiki M., Birembaut P., and Thompson E.W. (1997). Implication of collagen type I-induced membrane-type 1-matrix metalloproteinase expression and matrix metalloproteinase-2 activation in the metastatic progression of breast carcinoma. Lab Invest. 76:651–60.

Gomis-Ruth F.X., Maskos K., Betz M., Bergner A., Huber R., Suzuki K., Yoshida N., Nagase H., Brew K., Bourenkov G.P., Bartunik H., and Bode W. (1997). Mechanism of inhibition of the human matrix metalloproteinase stromelysin-1 by TIMP-1. Nature 389:77–81.

Graesser D., Mahooti S., Haas T., Davis S., Clark R.B., and Madri J.A. (1998). The interrelationship of alpha4 integrin and matrix metalloproteinase-2 in the pathogenesis of experimental autoimmune encephalomyelitis. Lab Invest. 78:1445–58.

Greene J., Wang M., Liu Y.E., Raymond L.A., Rosen C., and Shi Y.E. (1996). Molecular cloning and characterization of human tissue inhibitor of metalloproteinase 4. J Biol Chem. 271:30375–80.

Hershkoviz R., Goldkorn I., and Lider O. (1995). Tumour necrosis factor-alpha interacts with laminin and functions as a pro-adhesive cytokine. Immunology 85:125–30.

Himelstein B.P., Canete-Soler R., Bernhard E.J., and Muschel R.J. (1994). Induction of fibroblast 92 kDa gelatinase/type IV collagenase expression by direct contact with metastatic tumor cells. J Cell Sci. 107:477–86.

Hua J., and Muschel R.J. (1996). Inhibition of matrix metalloproteinase 9 expression by a ribozyme blocks metastasis in a rat sarcoma model system. Cancer Res. 56:5279–84.

Itoh Y., and Nagase H. (1995). Preferential inactivation of tissue inhibitor of metalloproteinases-1 that is bound to the precursor of matrix metalloproteinase 9 (progelatinase B) by human neutrophil elastase. J Biol Chem. 270:16518–21.

Karelina T.V., Hruza G.J., Goldberg G.I., and Eisen A.Z. (1993). Localization of 92-kDa type IV collagenase in human skin tumors: comparison with normal human fetal and adult skin. J Invest Dermatol. 100:159–65.

Kossakowska A.E., Edwards D.R., Lee S.S., Urbanski L.S., Stabbler A.L., Zhang C.L., Phillips B.W., Zhang Y., and Urbanski S.J. (1998). Altered balance between matrix metalloproteinases and their inhibitors in experimental biliary fibrosis. Am J Pathol. 153:1895–902.

Kruger A., Fata J.E., and Khokha R. (1997). Altered tumor growth and metastasis of a T-cell lymphoma in Timp-1 transgenic mice. Blood 90:1993–2000.

Kruger A., Sanchez-Sweatman O.H., Martin D.C., Fata J.E., Ho A.T., Orr F.W., Ruther U., and Khokha R. (1998). Host TIMP-1 overexpression confers resistance to experimental brain metastasis of a fibrosarcoma cell line. Oncogene 16:2419–23.

Leco K.J., Khokha R., Pavloff N., Hawkes S.P., and Edwards D.R. (1994). Tissue inhibitor of metalloproteinases-3 (TIMP-3) is an extracellular matrix-associated protein with a distinctive pattern of expression in mouse cells and tissues. J Biol Chem. 269:9352–60.

Leco K.J., Apte S.S., Taniguchi G.T., Hawkes S.P., Khokha R., Schultz G.A., and Edwards D.R. (1997). Murine tissue inhibitor of metalloproteinases-4 (Timp-4): cDNA isolation and expression in adult mouse tissues. FEBS Lett. 401:213–7.

Leppert D., Hauser S.L., Kishiyama J.L., An S., Zeng L., and Goetzl E.J. (1995). Stimulation of matrix metalloproteinase-dependent migration of T cells by eicosanoids. FASEB J. 9:1473–81.

Leppert D., Waubant E., Burk M.R., Oksenberg J.R., and Hauser S.L. (1996). Interferon beta-1b inhibits gelatinase secretion and in vitro migration of human T cells: a possible mechanism for treatment efficacy in multiple sclerosis. Ann Neurol. 40:846–852.

Leppert D., Ford J., Stabler G., Grygar C., Lienert C., Huber S., Miller K.M., Hauser S.L., and Kappos L. (1998). Matrix metalloproteinase-9 (gelatinase B) is selectively elevated in CSF during relapses and stable phases of multiple sclerosis. Brain 121:2327–34.

Mach F., Schonbeck U., Fabunmi R.P., Murphy C., Atkinson E., Bonnefoy J.Y., Graber P., and Libby P. (1999). T lymphocytes induce endothelial cell matrix metalloproteinase expression by a CD40L-dependent mechanism: implications for tubule formation. Am J Pathol. 154:229–38.

Malik N., Greenfield B.W., Wahl A.F., and Kiener P.A. (1996). Activation of human monocytes through CD40 induces matrix metalloproteinases. J Immunol. 156:3952–60.

Masson R., Lefebvre O., Noel A., Fahime M.E., Chenard M.P., Wendling C., Kebers F., LeMeur M., Dierich A., Foidart J.M., Basset P., and Rio M.C. (1998). In vivo evidence that the stromelysin-3 metalloproteinase contributes in a paracrine manner to epithelial cell malignancy. J Cell Biol. 140:1535–41.

Massova I., Kotra L.P., Fridman R., and Mobashery S. (1998). Matrix metalloproteinases: structures, evolution, and diversification. FASEB J. 12:1075–95.

Matsumoto S., Katoh M., Saito S., Watanabe T., and Masuho Y. (1997). Identification of soluble type of membrane-type matrix metalloproteinase-3 formed by alternatively spliced mRNA. Biochim Biophys Acta. 1354:159–70.

Miyagi E., Yasumitsu H., Hirahara F., Nagashima Y., Minaguchi H., Miyazaki K., and Umeda M. (1995). Marked induction of gelatinases, especially type B, in host fibroblasts by human ovarian cancer cells in athymic mice. Clin Exp Metastasis. 13:89–96.

Okada Y., Watanabe S., Nakanishi I., Kishi J., Hayakawa T., Watorek W., Travis J., and Nagase H. (1988). Inactivation of tissue inhibitor of metalloproteinases by neutrophil elastase and other serine proteinases. FEBS Lett. 229:157–60.

Parsons S.L., Watson S.A., and Steele R.J. (1997). Phase I/II trial of batimastat, a matrix metalloproteinase inhibitor, in patients with malignant ascites. Eur J Surg Oncol. 23:526–31.

Pei D. (1999). Identification and characterization of the fifth membrane-type matrix metalloproteinase MT5-MMP. J Biol Chem. 274:8925–32.

Powell W.C., Knox J.D., Navre M., Grogan T.M., Kittelson J., Nagle R.B., and Bowden G.T. (1993). Expression of the metalloproteinase matrilysin in DU-145 cells increases their invasive potential in severe combined immunodeficient mice. Cancer Res. 53:417–22.

Powell W.C., and Matrisian L.M. (1997). Complex role of Matrix metalloproteinases in tumor progression. In Attempts to Understand Matastasis Formation, Vol. I, Gunther, U., and Birchmeier, W., Eds. (Springer), pp. 1–22.

Primrose J.N., Bleiberg H., Daniel F., Van Belle S., Mansi J.L., Seymour M., Johnson P.W., Neoptolemos J.P., Baillet M., Barker K., Berrington A., Brown P.D., Millar A.W., and Lynch K.P. (1999). Marimastat in recurrent colorectal cancer: exploratory evaluation of biological activity by measurement of carcinoembryonic antigen. Br J Cancer. 79:509–14.

Proost P., Van Damme J., and Opdenakker G. (1993). Leukocyte gelatinase B cleavage releases encephalitogens from human myelin basic protein. Biochem Biophys Res Commun. 192: 1175–1181.

Puyraimond A., Weitzman J.B., Babiole E., and Menashi S. (1999). Examining the relationship between the gelatinolytic balance and the invasive capacity of endothelial cells. J Cell Sci. 112:1283–90.

Pyke C., Ralfkiaer E., Huhtala P., Hurskainen T., Dano K., and Tryggvason K. (1992). Localization of messenger RNA for Mr 72,000 and 92,000 type IV collagenases in human skin cancers by in situ hybridization. Cancer Res. 52:1336–41.

Rajavashisth T.B., Liao J.K., Galis Z.S., Tripathi S., Laufs U., Tripathi J., Chai N.N., Xu X.P., Jovinge S., Shah P.K., and Libby P. (1999). Inflammatory cytokines and oxidized low density lipoproteins increase endothelial cell expression of membrane type 1-matrix metalloproteinase. J Biol Chem. 274:11924–9.

Romanic A.M., and Madri J.A. (1994). The induction of 72-kD gelatinase in T cells upon adhesion to endothelial cells is VCAM-1 dependent. J Cell Biol. 125:1165–78.

Sato H., Takino T., Okada Y., Cao J., Shinagawa A., Yamamoto E., and Seiki M. (1994). A matrix metalloproteinase expressed on the surface of invasive tumour cells. Nature 370:61–5.

Schonbeck U., Mach F., Sukhova G.K., Murphy C., Bonnefoy J.Y., Fabunmi R.P., and Libby P. (1997). Regulation of matrix metalloproteinase expression in human vascular smooth muscle cells by T lymphocytes: a role for CD40 signaling in plaque rupture? Circ Res. 81:448–54.

Sehgal G., Hua J., Bernhard E.J., Sehgal I., Thompson T.C., and Muschel R.J. (1998). Requirement for matrix metalloproteinase-9 (gelatinase B) expression in metastasis by murine prostate carcinoma. Am J Pathol. 152:591–6.

Shofuda K., Yasumitsu H., Nishihashi A., Miki K., and Miyazaki K. (1997). Expression of three membrane-type matrix metalloproteinases (MT-MMPs) in rat vascular smooth muscle cells and characterization of MT3-MMPs with and without transmembrane domain. J Biol Chem. 272:9749–54.

Silbiger S.M., Jacobsen V.L., Cupples R.L., and Koski R.A. (1994). Cloning of cDNAs encoding human TIMP-3, a novel member of the tissue inhibitor of metalloproteinase family. Gene 141:293–7.

Strongin A.Y., Collier I., Bannikov G., Marmer B.L., Grant G.A., and Goldberg G.I. (1995). Mechanism of cell surface activation of 72-kDa type IV collagenase. Isolation of the activated form of the membrane metalloprotease. J Biol Chem. 270:5331–8.

Stuve O., Dooley N.P., Uhm J.H., Antel J.P., Francis G.S., Williams G., and Yong V.W. (1996). Interferon beta-1b decreases the migration of T lymphocytes in vitro: effects on matrix metalloproteinase-9. Ann Neurol. 40:853–863.

Sun Y., Hegamyer G., Kim H., Sithanandam K., Li H., Watts R., and Colburn N.H. (1995). Molecular cloning of mouse tissue inhibitor of metalloproteinases-3 and its promoter. Specific lack of expression in neoplastic JB6 cells may reflect altered gene methylation. J Biol Chem. 270:19312–9.

Tsunezuka Y., Kinoh H., Takino T., Watanabe Y., Okada Y., Shinagawa A., Sato H., and Seiki M. (1996). Expression of membrane-type matrix metalloproteinase 1 (MT1-MMP) in tumor cells enhances pulmonary metastasis in an experimental metastasis assay. Cancer Res. 56:5678–83.

Valente P., Fassina G., Melchiori A., Masiello L., Cilli M., Vacca A., Onisto M., Santi L., Stetler-Stevenson W.G., and Albini A. (1998). TIMP-2 over-expression reduces invasion and angiogenesis and protects B16F10 melanoma cells from apoptosis. Int J Cancer. 75:246–53.

Vu T.H., Shipley J.M., Bergers G., Berger J.E., Helms J.A., Hanahan D., Shapiro S.D., Senior R.M., and Werb Z. (1998). MMP-9/gelatinase B is a key regulator of growth plate angiogenesis and apoptosis of hypertrophic chondrocytes. Cell 93:411–22.

Wang M., Liu Y.E., Greene J., Sheng S., Fuchs A., Rosen E.M., and Shi Y.E. (1997). Inhibition of tumor growth and metastasis of human breast cancer cells transfected with tissue inhibitor of metalloproteinase 4. Oncogene 14:2767–74.

Wassenaar A., Verschoor T., Kievits F., Den Hartog M.T., Kapsenberg M.L., Everts V., and Snijders A. (1999). CD40 engagement modulates the production of matrix metalloproteinases by gingival fibroblasts. Clin Exp Immunol. 115:161–7.

Weeks B.S., Schnaper H.W., Handy M., Holloway E., and Kleinman H.K. (1993). Human T lymphocytes synthesize the 92 kDa type IV collagenase (gelatinase B). J Cell Physiol. 157:644–9.

Willmroth F., Peter H.H., and Conca W. (1998). A matrix metalloproteinase gene expressed in human T lymphocytes is identical with collagenase 3 from breast carcinomas. Immunobiology. 1998 Feb; 198(4):375–84.

Wize J., Sopata I., Smerdel A., and Maslinski S. (1998). Ligation of selectin L and integrin CD11b/CD18 (Mac-1) induces release of gelatinase B (MMP-9) from human neutrophils. Inflamm Res. 47:325–7.

Wojtowicz-Praga S., Low J., Marshall J., Ness E., Dickson R., Barter J., Sale M., McCann P., Moore J., Cole A., and Hawkins M.J. (1996). Phase I trial of a novel matrix metalloproteinase inhibitor batimastat (BB-94) in patients with advanced cancer. Invest New Drugs. 14:193–202.

Xia M., Leppert D., Hauser S.L., Sreedharan S.P., Nelson P.J., Krensky A.M., and Goetzl E.J. (1996). Stimulus specificity of matrix metalloproteinase dependence of human T cell migration through a model basement membrane. J. Immunol. 156:160–7.

Yao P.M., Maitre B., Delacourt C., Buhler J.M., Harf A., and Lafuma C. (1997). Divergent regulation of 92-kDa gelatinase and TIMP-1 by HBECs in response to IL-1beta and TNF-alpha. Am J Physiol. 273:L866–74.

Zhou H., Bernhard E.J., Fox F.E., and Billings P,C. (1993). Induction of metalloproteinase activity in human T-lymphocytes. Biochim Biophys Acta. 1177:174–8.

